# 1-Tx/5-Rx Through-Wall UWB Switched-Antenna-Array Radar for Detecting Stationary Humans

**DOI:** 10.3390/s20236828

**Published:** 2020-11-29

**Authors:** Artit Rittiplang, Pattarapong Phasukkit

**Affiliations:** School of Engineering, King Mongkut’s Institute of Technology Ladkrabang, Bangkok 10520, Thailand; 59601306@kmitl.ac.th

**Keywords:** switched-antenna-array radar, Vivaldi antenna, UWB radar, linear antenna array, through-wall radar

## Abstract

This research proposes a through-wall S-band ultra-wideband (UWB) switched-antenna-array radar scheme for detection of stationary human subjects from respiration. The proposed antenna-array radar consists of one transmitting (Tx) and five receiving antennas (Rx). The Tx and Rx antennas are of Vivaldi type with high antenna gain (10 dBi) and narrow-angle directivity. The S-band frequency (2–4 GHz) is capable of penetrating non-metal solid objects and detecting human respiration behind a solid wall. Under the proposed radar scheme, the reflected signals are algorithmically preprocessed and filtered to remove unwanted signals, and 3D signal array is converted into 2D array using statistical variance. The images are reconstructed using back-projection algorithm prior to Sinc-filtered refinement. To validate the detection performance of the through-wall UWB radar scheme, simulations are carried out and experiments performed with single and multiple real stationary human subjects and a mannequin behind the concrete wall. Although the proposed method is an odd concept, the interest of this paper is applying the 1-Tx/5-Rx UWB switched-antenna array radar with the proposed method that is capable of distinguishing between the human subjects and the mannequin behind the concrete wall.

## 1. Introduction

Recent decades have witnessed the growth in adoption of ultra-wideband (UWB) radar technology to detect targets through walls. The advantages of through-the-wall UWB radars are greater penetration capability, higher resolution and azimuth discrimination, compared with continuous wave (CW) radars [1,2,3,4,5,6,7]. The UWB radar technology has been utilized in various applications, including security inspection, disaster rescue, and medicine. 

Modern radars rely on synthetic aperture radar (SAR) and multi-input-multi-output (MIMO) technologies to render high-resolution 2D/3D images of multiple targets, as opposed to monostatic radar technology with limited spatial resolution. SAR radars consist of one transmitting antenna (1-Tx) and one receiving antenna (1-Rx). In operation, either one or both antennas are motioned in parallel to the scanned object, and the received signal is spatially sampled in a regular grid. The technique produces high azimuth resolution but requires large-area scanning. The SAR technology are currently used in, e.g., ground penetrating radar (GPR), vehicle, rail, and airborne applications [8,9,10,11]. 

The MIMO technology is capable of rendering higher-resolution radar imaging. However, the technology suffers from delayed data acquisition due to multiple antennas [12,13,14,15,16,17,18,19,20,21,22]. MIMO radars are deployed for, e.g., breast cancer diagnosis and concealed weapon detection. Interestingly, a MIMO system with M transmitting elements and N receiving elements requires M × N virtual transceivers. In through-the-wall image radar, the MIMO technology can be applied for higher-resolution radar imaging, but suffers from delayed data acquisition due to multiple transmitting and multiple receiving during operation [12,13,14,15,16,17,18,19,20,21], and the large number of MIMO elements contributes to higher fabrication costs and complexity. As a result, references [23,24,25,26] proposed a UWB switched-antenna-array radar with low-cost fabrication of a 13-Tx/8-Rx structure that could achieve high performance in through-the-wall radar imaging systems, instead of the MIMO structure. Such a solution has become the subject of the paper to contribute to the downsizing of and increasing the quality of an S-band UWB switched-antenna-array radar from a 13-Tx/8-Rx to a 1-Tx/5-Rx radar, which provides lower cost fabrication and is enough to efficiently image human positions behind a concrete wall with the proposed methods. Advantages and benefits could be deployed in various through-obstruction applications, especially in hostage rescue operations. Note that the S-band frequency (2–4 GHz) is capable of penetrating non-metal solid objects and detecting human respirations behind the concrete wall [22,23,24,25,26]. Also, the transmitting (Tx) and receiving antennas (Rx1–Rx5) we designed are of Vivaldi type with high antenna gain (10 dBi) and directivity narrow-angle so that the antenna pattern can provide the imaging resolution, and the design parameters are easy with low-cost fabrication.

In the operation, the Tx antenna emits a UWB signal to the solid wall and human subjects and the Rx1–Rx5 antennas capture the reflected signals. The signals are algorithmically preprocessed and filtered to remove unwanted signals. The improved signal is 3D array and converted into 2D array using statistical variance analysis. A back-projection algorithm is subsequently implemented to reconstruct the images and is refined by the Sinc filter. In the study, simulations are carried out, and comparative experiments are conducted with single and multiple real stationary human subjects and a mannequin behind the concrete wall. In through-the-wall radar applications, heart rate detection is not important; respiration or movements of the person is enough to detect the lift.

The organization of this research is as follows: Section 1 is the introduction. Section 2 describes the Vivaldi antenna design and detection principle. Section 3 details the simulation results of the proposed UWB switched-antenna-array radar, and Section 4 discusses the experimental results with single and multiple real stationary human subjects and a mannequin behind the concrete wall. The concluding remarks are provided in Section 5.

## 2. Detection Principle

### 2.1. Vivaldi Antenna Design

**Figure 1:**
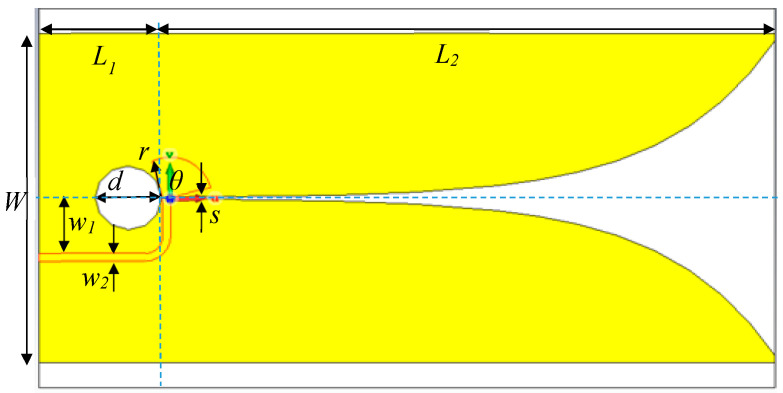
Geometry of the proposed transmitting (Tx) and receiving (Rx) Vivaldi antennas.

Figure 1 illustrates the proposed transmitting and receiving Vivaldi antennas (50 Ω input impedance) in S-band (2–4 GHz). In this research, the through-wall switched-antenna-array radar scheme consists of one transmitting (Tx) and five receiving (Rx) Vivaldi antennas. The advantages of Vivaldi antennas are high antenna gain (10 dBi), narrow-angle directivity, and ultra-wide frequency band [26,27,28,29,30,31,32,33]. Table 1 tabulates the dimensions of Tx and Rx Vivaldi antennas. 

In Figure 1, the antenna length (*L*_1_ and *L*_2_) is greater than half the wavelength (*λ*/2), and the width (*W*) is greater than a quarter wavelength (*λ*/4). A circular cavity diameter (*d*) is a quarter of the guide wavelength in the slot line (free space). The antenna is realized on FR-4 substrate whose dielectric constant permittivity (*ε_r_*), thickness, and loss tangent (*δ*) are 4.3, 1.6 mm, and 0.025. In the antenna design, the copper plate is initially etched into exponentially tapered profile according to Equation (1)

(1) $$y\left(x\right)=\frac{s}{2}e^{kx},\;\;\mathrm{where}\;\;k=\frac{1}{L_{2}}\ln\left(W/s\right)\;$$

**Figure 2:**
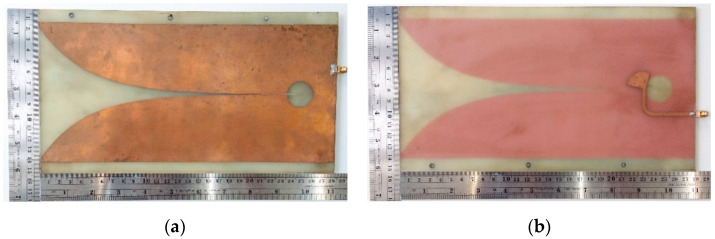
The Tx and Rx Vivaldi prototype antennas: (**a**) top view; (**b**) bottom view with the radiator.

Figure 2a,b respectively depict the top and bottom views of the Tx and Rx Vivaldi prototype antennas.

**Figure 3:**
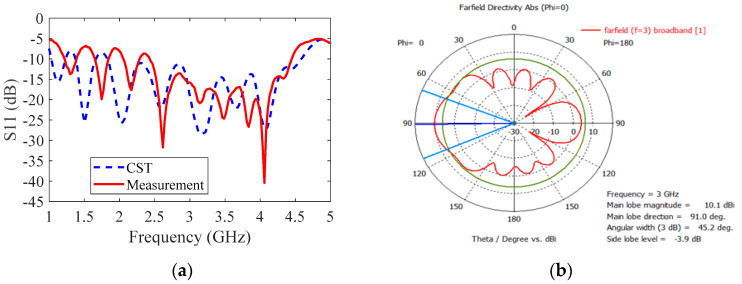
(**a**) comparison between the simulated and measured impedance matching (|S_11_| ≤ −10 dB) of Tx and Rx Vivaldi antennas; (**b**) Simulated radiation pattern of the proposed Vivaldi antenna at the center frequency of 3 GHz with a high gain of 10.1 dBi and angular width of 45.2°.

Figure 3a compares the simulated and measured impedance matching (|S_11_| ≤ −10 dB) Vivaldi antenna. The simulation was carried out using CST studio suite.

Figure 3 shows the designed antenna by the CST simulation. The frequency range of the reflection coefficient|S_11_| ≤ −10 dB is 1.8–4.5 GHz and the reflection coefficient of the fabricated antenna is 2.3–4.3 GHz. Also, the simulated radiation pattern at the center frequency of 3 GHz has a high gain of 10.1 dBi and angular width of 45.2° as shown in Figure 3b.

### 2.2. Vital Sign Model Behind the Wall

**Figure 4:**
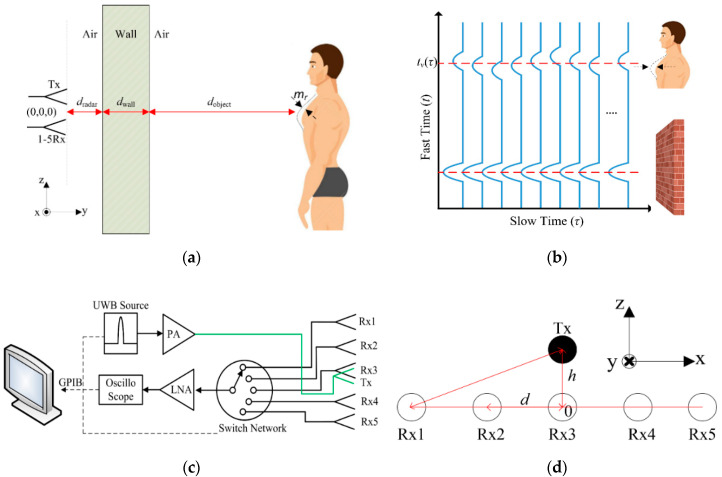
Principle of the through-the-wall ultra-wideband (UWB) radar: (**a**) round-trip Tx and Rx signals with human object; (**b**) raw data per channel; (**c**) block diagram of the 1-Tx/5-Rx UWB switched-antenna-array radar; (**d**) 1-Tx/5-Rx antennas arrangement with Rx3 at the origin.

The S-band UWB signal emitted from the Tx antenna penetrates efficiently through the concrete wall onto the human object and returns to a switched Rx antenna as shown in Figure 4a [22,23,24,25].

The reflected signals per one channel are measured in fast-time domain (wave-propagation time) with multiple overlapping slow-time measurements (time of signal detection) as shown in Figure 4b. In general, the received signals are modeled as the summation of multipath reflected signals from different objects with time delay [3], namely

(2) $$R_{n}\left(t,\tau \right)=\underset{p}{\sum }\sigma_{p}s\left(t-t_{p}\right)+\underset{o}{\sum }\sigma_{o}s\left(t-t_{o}\left(\tau \right)\right)+\underset{v}{\sum }\sigma_{v}s\left(t-t_{v}\left(\tau \right)\right)$$

with *nth* channel, *σ_p_*, *σ_o_*, *σ_v_* denotes stationary, non-stationary, and human objects, *s*(*t-t_n_*) is time-shifting of the transmitted signal, and *t* and *τ* are fast-time and slow-time domains [34,35,36,37,38,39]. $\sigma_{v}s\left(t-t_{v}\left(\tau \right)\right)$ denotes response due to the respiration movement time *t_v_*(*τ*), which is dependent on the respiration amplitude [6], namely

(3) $$t_{v}\left(\tau \right)=\frac{2}{c}\left(y_{0}\;+m_{r}\sin\left(2\pi f_{r}\tau \right)\right)$$

with

(4) $$y_{0}=d_{radar}+d_{wall}\sqrt{\varepsilon_{w}}+d_{object}$$

From Equation (3), *m_r_* and *f_r_* are movement amplitude and frequency of the respiration and *y_0_* is the nominal distance between the radar and the human chest. The *y_0_* lies on the *y*-axis and is used to estimate the human range, where *d_radar_*, *d_wall_*, and *d_object_* are the antenna-wall distance, wall thickness, and wall-human distance, ε_w_ is the wall permittivity which is assumed to be homogeneous, and *c* is the speed of light (*c* is the speed of light in vacuum).

The UWB antenna-array radar system consists of one transmitting antenna (1-Tx) and five receiving antennas (Rx1–Rx5), as shown in Figure 4c. To mitigate the coupling effect in the antenna array, the Tx antenna is stacked above the Rx array and vertically aligned with Rx3, as illustrated in Figure 4d. The signals from the human object are periodic which are attributable to the vital signs (i.e., respiration and heart rates), while those from the impenetrable object are not a function of time shift and thus are static as shown in Figure 4b. As a result, the slow time shift of reflected signals could be used to detect human physiological movements.

Given that the challenges of measurement of reflected signal in continuous time (Equation (2)) are quite complex, this research thus determines the reflected signal in discrete time [34,35,36,37,38,39]. *R_n_*[*k,l*] is the reflected signal of *nth* channel in discrete time

(5) $$R_{n}\left[k,l\right]=h_{n}\left[k,l\right]+c_{n}\left[k,l\right]+w_{n}\left[k,l\right]+q_{n}\left[k,l\right]$$

where *h_n_*[*k,l*], *c_n_*[*k,l*], *w_n_*[*k,l*], and *q_n_*[*k,l*] are the respiration signal, static signal, white noise, and non-static signal respectively, where *k* is discrete domain in fast time of *K* sampling and *l* is discrete domain in slow time of *L* sampling. Preprocessing is subsequently carried out to filter out unwanted signals (i.e., static signal, white noise, and non-static signal) and extract vital signs.

### 2.3. Preprocessing

The reflected signal in discrete time subsequently undergoes preprocessing to remove unwanted signals. The unwanted static signal is slow time-independent (Figure 4b) and can be removed by averaging l by the number of slow time sampling (L)

(6) $$\Omega_{n}\left[k,l\right]=R_{n}\left[k,l\right]-\frac{1}{L}\overset{L}{\underset{l=1}{\sum }}R_{n}\left[k,l\right]\;=h_{n}\left[k,l\right]+w_{n}\left[k,l\right]+q_{n}\left[k,l\right]$$

where *L* is the number of slow time sampling. The signal is further refined by linear least-squares

(7) $$W=\Omega -X{\left(X^{T}X\right)}^{-1}X^{T}\Omega$$

where *X* = [*x*_1_, *x*_2_], *x_1_* = [0,1,2,…*K*-1], *x_2_* = ${\left[1,1,1,\ldots ,1\right]}_{K}^{T}$ and the superscript *T* denotes transpose [36,37,38,39].

In practice, the first and second pulses of the received signal are respectively the antenna coupling and wall reflection. Singular value decomposition (SVD) has been proposed to address the issues. However, the SVD technique suffers from lengthy processing time due to the inverse matrix of large data. Given the inevitability of the antenna coupling and wall reflection at the minimum detectable range, this research deliberately designates the first and second pulses as zero [35,36,37].

(8) $$W\left[1:K_{zero},\;l\right]=0,\;\mathrm{where}\;\;K_{zero}=\mathrm{floor}\left[d_{min}/\Delta r\right]$$

where *d_min_* is the minimum detectable range and ∆*r* is the range resolution. 

The non-static signal *q*[*k,l*] and white noise *w*[*k,l*] are respectively reduced by using the smooth and Butterworth bandpass filters in fast and slow-time domains

(9) $$y\left[k,l\right]=\frac{1}{2F+1}\overset{F}{\underset{i=-F}{\sum }}W\left[k-i,l\right]$$

where *F* is the number of average data points on either side of *w*[*k,l*] and 2*F* + 1 is the span. The transfer function of Butterworth bandpass filter is written as

(10) $${\left|H\left(\omega \right)\right|}^{2}=\frac{1}{1+{\left(\omega /\omega_{c}\right)}^{2N_{f}}}$$

where *ω_c_* is the cutoff frequency and *N_f_* is the filter order and is set to 5 giving a good tradeoff between performance and complexity [38]. 

The fine-tuned received signal (with removal of unwanted signals) is rewritten as below. In reality, traces of white noise *w*_0_[*k,l*] and non-static signal *q*_0_[*k,l*] remain after the preprocessing:

(11) $$y_{n}\left[k,l\right]\approx h\left[k,l\right]+w_{0}\left[k,l\right]+q_{0}\left[k,l\right]$$

**Figure 5:**
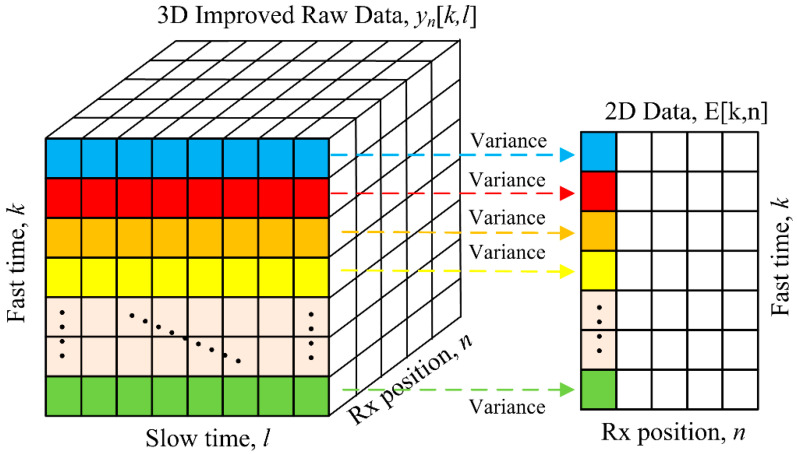
Conversion of the 3D data (Equation (11)) to the 2D data using statistical variance analysis.

It is a 3D array as shown on the left in Figure 5 consisting of fast time, slow time, and Rx position (Rx1–Rx5). Next, statistical variance analysis in Equation (12) is used to estimate the respiration movement from the 3D array (Equation (11)) providing a 2D array result illustrated on the right in Figure 5 [34,35,36,37], to prepare for the use of back projection (BP) method [40].

(12) $$E\left[k,n\right]=\frac{1}{L-1}\overset{L}{\underset{l=1}{\sum }}{\left[y_{n}\left[k,l\right]-\mu \right]}^{2}\;,\;\mathrm{where}\;\mu =\frac{1}{L}\overset{L}{\underset{l=1}{\sum }}y_{n}\left[k,l\right]$$

### 2.4. Back Projection Method

In radar image processing, there are three approaches: range-doppler (RD), wavenumber domain (ω-k), and space-time domain. The first and second approaches are fast Fourier transform (FFT)-based techniques that require equally-spaced data and quantification of the velocity change between different mediums despite high-resolution images [40]. As a result, this research relies on the space-time domain as a function of time shift, using the BP algorithm. Given *E*[*k,n*] in Equation (12), the image can be reconstructed by BP algorithm (Equation (13)), where ∆*t* is fast time resolution

(13) $$I\left[x,y\right]=\overset{N}{\underset{n=1}{\sum }}E\left[k_{\mathrm{index}},n\right],\;\;\mathrm{where}\;k_{\mathrm{index}}=\mathrm{floor}\left[\frac{t_{\mathrm{index}}}{\Delta t}\right]$$

and

(14) $$t_{\mathrm{index}}=\frac{\sqrt{y^{2}+{\left(x-x_{Tx}\right)}^{2}}+\sqrt{y^{2}+{\left(x-x_{Rx}\right)}^{2}}}{c}$$

where *t*_index_ is the time index of fast time domain, *x* and *y* are the cross-range and down-range of the image grid.

The post-BP reconstructed image *I*[*x*,*y*] suffers from the shadowing effect. Several techniques have been proposed to mitigate the shadowing effect [6,19]. In this research, the Sinc filter [40] is simple and enough utilized to suppress the shadowing effect in the reconstructed image $\hat{I}\left[x,y\right]$

(15) I^x,y=ReIFFTSf,y.Hf

where

(16) $$S\left(f,y\right)=FFT_{x}\left\{I\left[x,y\right]\right\}\;\;\mathrm{and}\;\;H\left(f\right)=\left|4\sin\left(2\pi f\right)\right|sinc^{2}\left(2f\right)$$

**Figure 6:**
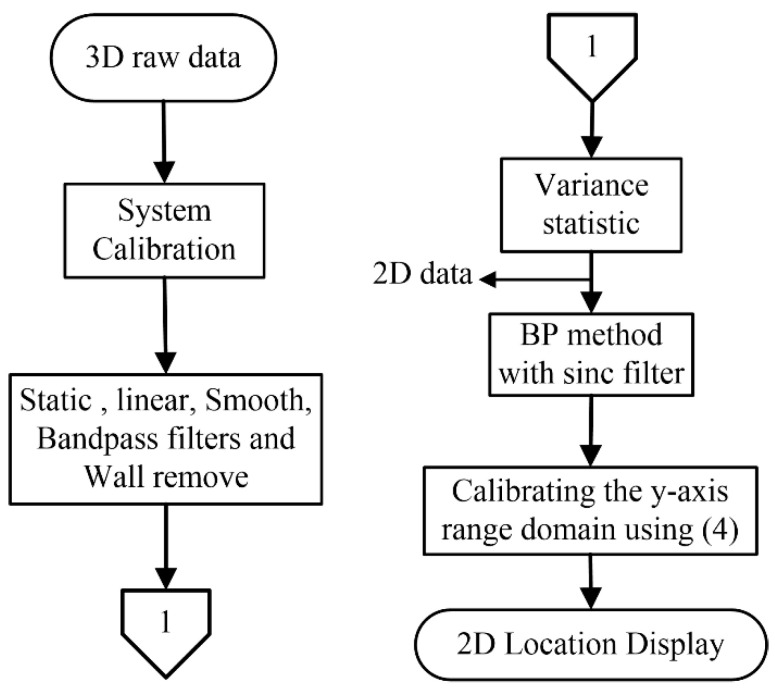
Flowchart of the proposed method.

In essence, this research proposes a through-wall S-band UWB switched-antenna-array radar scheme, consisting of one transmitting (1-Tx) and five receiving antennas (5-Rx), enough to efficiently detect multiple stationary human subjects from respiration. The S-band frequency (2–4 GHz) is capable of penetrating non-metal solid objects and detecting human respiration behind the concrete wall [6,22,23,24,25,26]. In the operation, the reflected signals are preprocessed to remove unwanted signals using filters and algorithmic scheme. Statistical variance analysis is undertaken to convert 3D to 2D signal array. Back-projection algorithm is implemented to reconstruct the images and is further refined by the Sinc filter algorithm for through-the-wall radar images of human subjects. A flow chart is illustrated in Figure 6 below. The system calibration means that the reflected signal of each channel must be calibrated to compensate for time-shift delay lost due to transmission line and microwave devices and the time-shift delay loss directly affects the image quality. In the case of no human, we can basically observe the slow time of pulses on the first receiving antenna, or make sure of the human position by computing the variance statistic of the first receiving antenna.

## 3. Simulation Result

**Figure 7:**
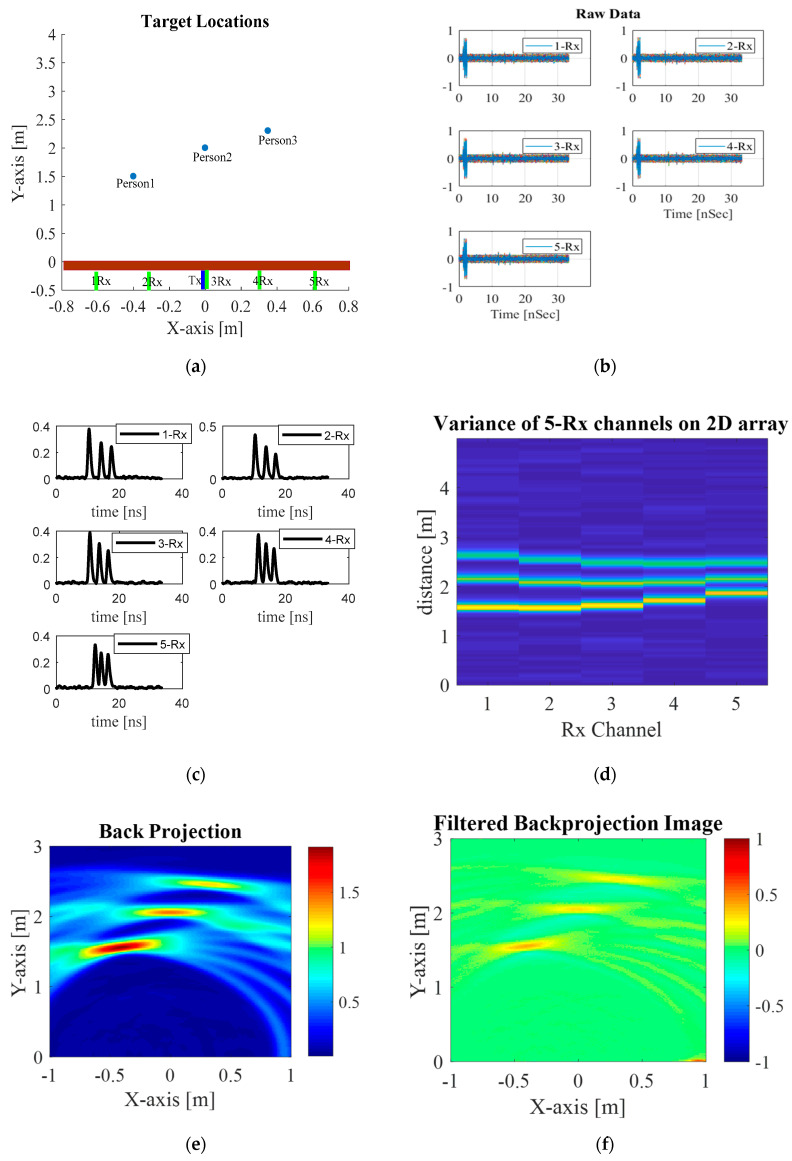
Simulation conditions and results: (**a**) three stationary human subjects behind the wall, (**b**) simulated received signals carrying vital signs of three human subjects, (**c**) statistical variance analysis of each channel, (**d**) statistical variance analysis of each channel on 2D data, (**e**) radar imaging by back projection, (**f**) refined radar imaging using Sinc-filtered back projection, given −1 < x < 1 and 0 < y < 3.

Simulations were carried out with three stationary human subjects behind the solid wall. The coordinates (locations) of the three human subjects are (−0.4,1.5), (0,2), and (0.32,2.4) meter units and the solid wall is at y = 0, as shown in Figure 7a. The respiration rates are assumed as a periodical sinusoidal displacement of 1.8 mm [3] and a frequency of 0.4 Hz, given the respiration rate of 24 breaths per minute. The transmit signal is of the first-order Gaussian, following [3,35,36], with 3 GHz center frequency and the antenna-array center (Rx3) positioned at the origin (0,0). The space between receiving antennas (Rx1–Rx5) is 30 cm. Table 2 tabulates the simulation parameters of the through-wall UWB switched-antenna-array radar for detection of human subjects.

Figure 7b shows the simulated received signals of Rx1–Rx5 carrying the vital signs of three stationary human subjects. The Rx data are 3D array converted into 2D array data using Equation (12), as shown in Figure 7c and plotted on a 2D display in Figure 7d. The data having a parabola shape is attributable to the switch positions of the Rx antennas. Next, the data is reconstructed using back-projection algorithm. The results are illustrated in Figure 7e and the image is further refined by the Sinc filter, resulting in Figure 7f.

## 4. Radar System Implementation

**Figure 8:**
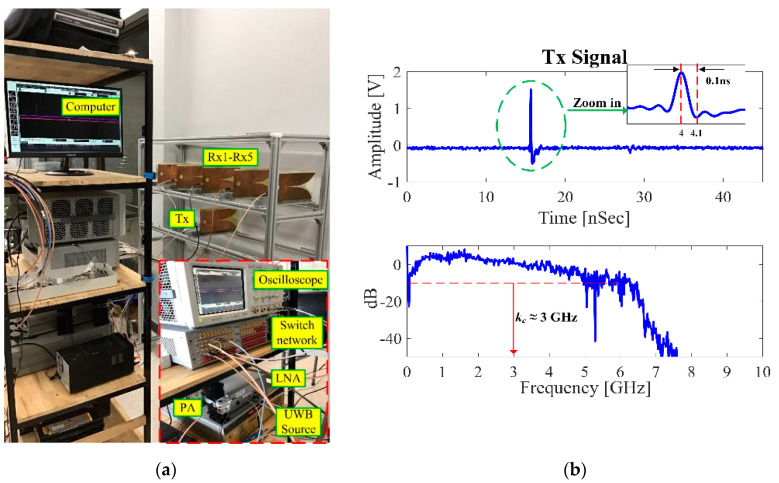
(**a**) Experimental setup of the switched-antenna-array radar prototype. The space between receiving antennas (Rx1–Rx5) is 30 cm; (**b**) the UWB signal of the transmitting antenna (Tx).

The through-wall UWB switched-antenna-array radar used for this experiment is shown in Figure 8a referred to Figure 4c, consisting of one transmitting antenna (1-Tx) and five receiving antennas (5-Rx) connected to the switch network. The experimental components were listed in Table 3. 

The transmitting antenna (Tx) and the receiving-antennas array (Rx1–Rx5) are physically separated by stacking Tx above the Rx array (with a distance of 5 cm) to mitigate the Tx-to-Rx coupling effect. The space between receiving antennas (Rx1–Rx5) is 30 cm. 

Figure 8b illustrates the UWB signal of Tx with the center frequency of 3 GHz, covering the bandwidth frequency of 5.2 GHz. The bandwidth does not fully follow the FCC regulations of 3.1–10.6 GHz in that the reference level is set to limit interference to existing communication systems only. This system operated at the S-band (2–4GHz) to maintain both high-spatial-resolution and wall-penetrating; it is capable of penetrating non-metal solid objects and detecting human respirations behind the concrete wall [22,23,24,25,26]. The transmitted power on the chest surface does not exceed 10 W/m^2^ (permissible exposure limit) so electromagnetic radiation poses no safety threat.

In this system, a computer with GPIB pulses the UWB source, controls the switch network, retrieves data from the oscilloscope, processes the data, and displays the radar image. The peak transmitted power was about 10 dBm, the detectable range of this system was approximately 5.25 m, and we can increase the detection range by increasing the pulse repetition interval (PRI), but the expense of more detection time. The slow-time signals per one receiving were captured approximately at the rate of 256 times in 60 s (under Nyquist sampling) to accurately detect respiration rate. For five receiving the UWB imaging system took the detection time of approximately 5 min. For each slow-time measurement, the received signals were measured throughout 35 ns (fast time), which is then discretized to 7985 points. The switch-network output is amplified by the low-noise amplifier (LNA) and then fed into the oscilloscope to capture the reflected signal. In the signal processes, the reflected signal of each channel must be calibrated to compensate for time-shift delay lost due to transmission line and microwave devices, then preprocessed to remove unwanted signals, and statistical variance analysis is undertaken to convert 3D to 2D array. Back-projection algorithm is implemented to reconstruct the image from a 2D array and further refined by the Sinc filter. The step-by-step of the proposed method is denoted in Figure 6. This system with the proposed methods took a total time of 5.45 min to both image and distinguish between human and non-human subjects. In the case of human motion detection, this system can detect it by basically observing the time-shift delay of a received signal.

To validate the detection performance of the proposed UWB switched-antenna-array radar, experiments are conducted with single and multiple real stationary human subjects behind the concrete wall. All volunteers signed the informed consent and were given instructions about the experiment and attached contact sensors before measurements.

### 4.1. Through-the-Wall Detection of Single Stationary Human

**Figure 9:**
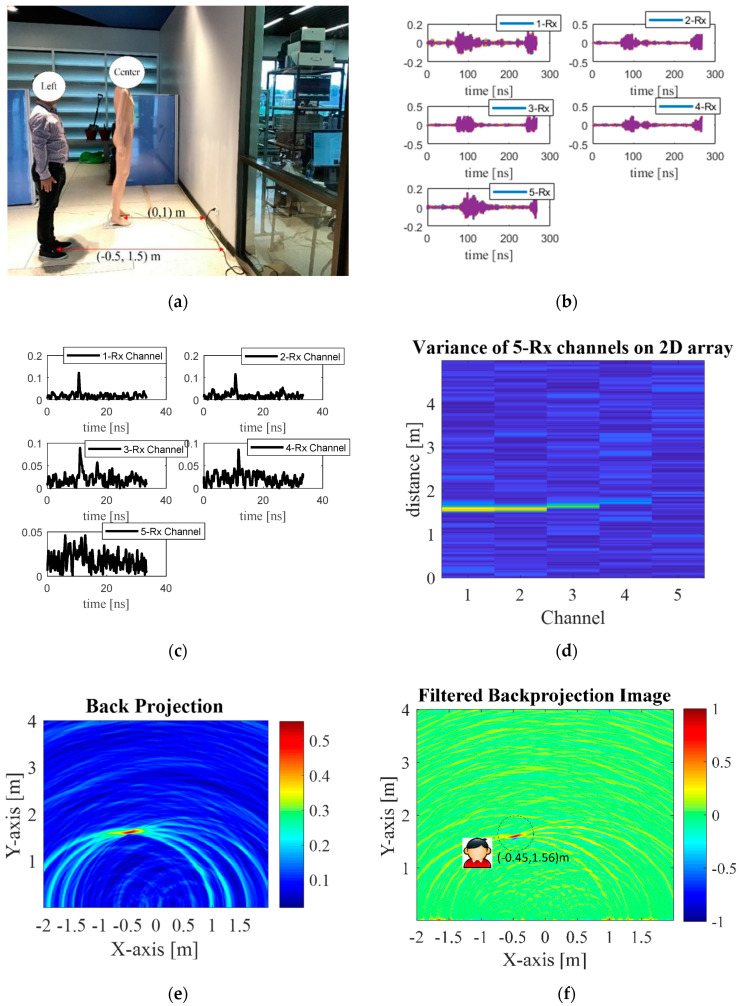
Through-the-wall detection of one human subject at (−0.5 m, 1.5 m) and a mannequin at (0, 1 m): (**a**) the experimental scene with real human and mannequin; (**b**) raw data of each channel; (**c**) statistical variance analysis of each channel, (**d**) statistical variance analysis of each channel on 2D display, (**e**) radar imaging by back projection, (**f**) refined radar imaging using Sinc-filtered back projection.

Figure 9a depicts the experimental scene with a stationary real human subject and a mannequin. The locations (coordinates) of the stationary real human subject and the mannequin are (−0.5 m, 1.5 m) and (0, 1 m).

The Rx raw data of each channel are shown in Figure 9b and pre-processed with the proposed method shown in Figure 9c,d (display 2D). Next, the 2D array is reconstructed using back-projection algorithm (Figure 9e) and further refined by the Sinc filter (Figure 9f). The UWB switched-antenna-array radar with the proposed method is capable of distinguishing between the human subject and the mannequin behind the concrete wall. Nevertheless, the proposed through-wall radar exhibits small down-range and cross-range estimation errors of 4% and 10% due to the round-trip delays in the wall and the antenna fabrication.

### 4.2. Through-the-Wall Detection of Multiple Stationary Humans

**Figure 10:**
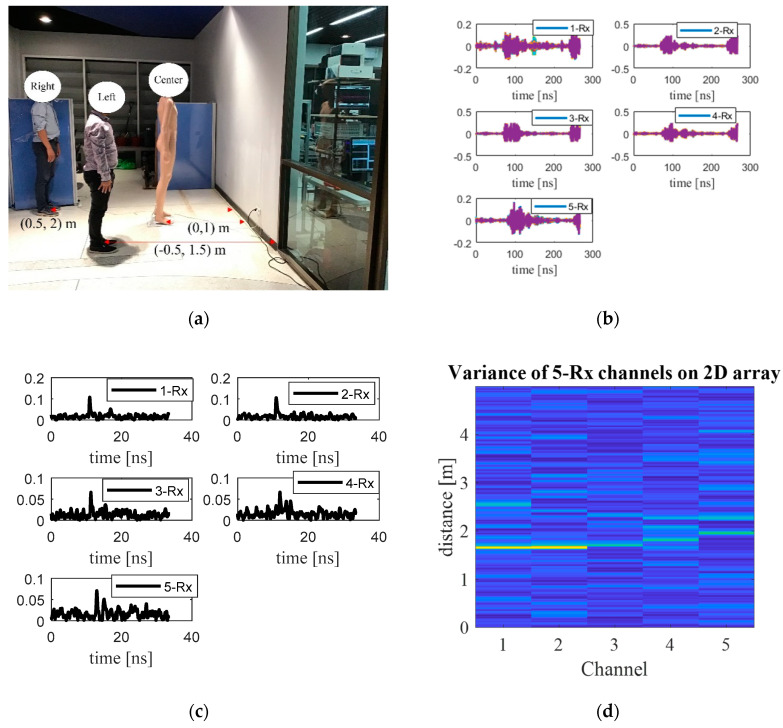

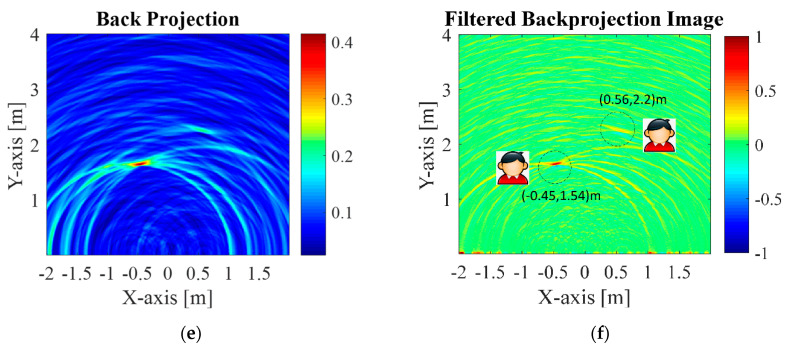
Through-the-wall detection of two human subjects at (−0.5 m, 1.5 m) and (0.5 m, 2 m) and a mannequin at (0, 1 m): (**a**) the experimental scene with two real humans and mannequin, (**b**) raw data of each channel; (**c**) statistical variance analysis of each channel, (**d**) statistical variance analysis of each channel on 2D display, (**e**) radar imaging by back projection, (**f**) refined radar imaging using Sinc-filtered back projection.

Figure 10a shows the experimental scene with two stationary real human subjects and a mannequin. The locations (coordinates) of the first human subject, the mannequin, and the second human subject are (−0.5 m, 1.5 m), (0, 1 m), and (0.5 m, 2 m). 

The Rx raw data of each channel are shown in Figure 10b and processed with the proposed method resulting in Figure 10c,d (display 2D). Next, the 2D array is reconstructed using back-projection algorithm (Figure 10e) and further refined by the Sinc filter (Figure 10f). The through-wall UWB switched-antenna-array radar with the proposed method is capable of distinguishing between both human subjects and the mannequin. The down-range and cross-range estimation errors are similar to those of the single stationary human, independent of the number of human subjects. More measurements were taken with different locations as shown below.

**Figure 11:**
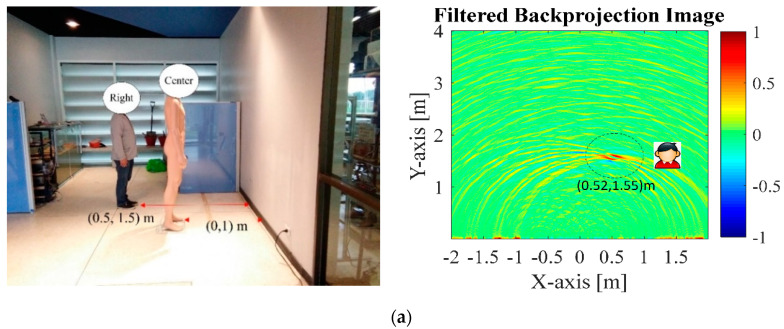

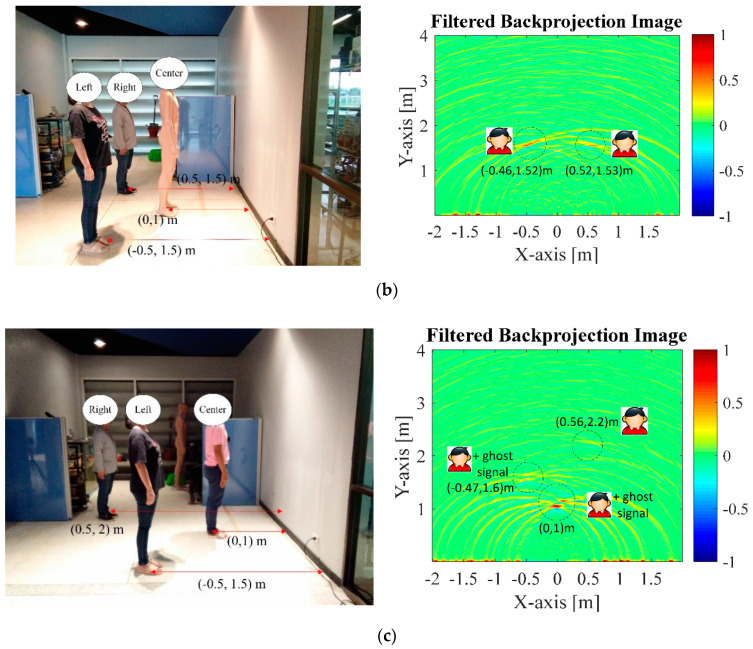
Through-the-wall detection: (**a**) a human (0.5 m, 1.5 m) and a mannequin (0, 1 m); (**b**) two humans (−0.5 m, 1.5 m) and (0.5 m, 1.5 m) a mannequin (0, 1 m); (**c**) three humans at (−0.5 m, 1.5 m), (0, 1 m) and (0.5 m, 2 m).

The experimental results were in good agreement, distinguishing between both human subjects and the mannequin behind the concrete wall, but it is probably difficult to remove the ghost signal, especially in Figure 11c, due to the reflection of the electromagnetic wave scattering off the objects. Although the proposed method is an odd concept, the interest of this paper is applying the 1-Tx/5-Rx through-wall UWB switched-antenna array radar for imaging stationary humans behind the concrete wall.

## 5. Conclusions

This research proposes a through-wall S-band 1-Tx/5-Rx UWB switched-antenna-array radar scheme for detection of stationary human subjects from respiration. The transmitting (Tx) and receiving antennas (Rx) are of Vivaldi type with high antenna gain (10 dBi) and narrow-angle directivity. In operation, the Tx antenna emits a UWB signal to the solid wall and human subjects and the Rx1–Rx5 antennas capture the reflected signals. The signals are algorithmically preprocessed to remove unwanted signals, and statistical variance analysis is undertaken to convert 3D to 2D array. Back-projection algorithm is implemented to reconstruct the images and refined by the Sinc filter. In the study, simulations were carried out, and experiments were performed with single and multiple real stationary human subjects and a mannequin behind the concrete wall. The results reveal that the through-wall UWB switched-antenna-array radar can distinguish between the human subjects and the mannequin. With minor modifications, the proposed through-wall radar scheme can efficiently be deployed in rescue operations during natural disasters.

## Figures and Tables

**Table 1:** Dimensions of Tx and Rx Vivaldi antennas for S-band frequency (2–4 GHz).

Parameter	Value	Parameter	Value
*W*	130 mm	*ε_r_* (FR4)	4.3
*L*_1_	50 mm	*s*	0.5 mm
*L*_2_	230 mm	*d*	25 mm
*w*_1_	15 mm	*r*	16 mm
*w_2_*	3 mm	*θ*	90°
PCB thickness	1.6 mm	copper thickness	0.035 mm

**Table 2:** Simulation parameters of the through-the-wall UWB switched-antenna-array radar.

Parameter	Description	Value
A_0_	Tx voltage	1 V
*k*_c_	Central frequency	3 GHz
f_s_fast_	Sampling frequency in fast time	20 GHz
PRI	Pulse repetition interval	35 ns
τ_max_	Maximum slow time	60 s
f_s_slow_	Sampling frequency in slow time	50 Hz

**Table 3:** Descriptions of the experimental hardware.

Hardware	Brand/Model/Type	Specification
UWB source	HP-8133A pulse generator	1.0 V Peak voltage, Central frequency 3 GHz, BW = 0.2‒5.9 GHz
Tx and Rx antennas	Vivaldi type	S-Band at 9–10 dBi, angular ≈ 45°
Power amplifier (PA)	ZVE-8G+ Mini-Circuits	2–8 GHz, 30 dBm
Low noise amplifier (LNA)	R and K-AA260-OS	2–5 GHz, 26 dBm
Analog-to-digital (ADC)	Oscilloscope, Agilent Infinium DSO80604B	Maximum frequency of 6 GHz, Sampling rate 40 GSa/s
RF-Switch	Agilent 34980A	Multifunction switch network
GPIB USB port	Agilent GPIB, 82357B	Transfer rate of 850 KB/sec
Wall	Concrete	4-inch thickness, Permittivity (ε_r_) of 4.5
